# Effects of a 6-month multimodal training intervention on retention of functional fitness in older adults: A randomized-controlled cross-over design

**DOI:** 10.1186/1479-5868-9-107

**Published:** 2012-09-10

**Authors:** Janus Gudlaugsson, Vilmundur Gudnason, Thor Aspelund, Kristin Siggeirsdottir, Anna S Olafsdottir, Palmi V Jonsson, Sigurbjorn A Arngrimsson, Tamara B Harris, Erlingur Johannsson

**Affiliations:** 1 Centre for Research in Sport and Health Sciences, University of Iceland, Laugarvatn, Iceland; 2 Icelandic Heart Association, Reykjavik, Iceland; 3 Faculty of Medicine, University of Iceland, Reykjavik, Iceland; 4 Department of Geriatrics, Landspitali – University Hospital, Reykjavik, Iceland; 5 Laboratory of Epidemiology, Demography and Biometry, Intramural Research Program, National Institute of Aging, Baltimore, MD, USA

**Keywords:** Physical activity, Functional fitness, SPPB, 6 MW, Strength, Cross-over design

## Abstract

**Background:**

Older adults have the highest rates of disability, functional dependence and use of healthcare resources. Training interventions for older individuals are of special interest where regular physical activity (PA) has many health benefits. The main purpose of this study was to assess the immediate and long-term effects of a 6-month multimodal training intervention (MTI) on functional fitness in old adults.

**Methods:**

For this study, 117 participants, 71 to 90 years old, were randomized in immediate intervention group and a control group (delayed intervention group). The intervention consisted of daily endurance and twice-a-week strength training. The method was based on a randomized-controlled cross-over design. Short Physical Performance Battery (SPPB), 8 foot up-and-go test, strength performance, six min walking test (6 MW), physical activity, BMI and quality of life were obtained at baseline, after a 6-month intervention- and control phase, again after 6-month crossover- and delayed intervention phase, and after anadditional 6-month follow-up.

**Results:**

After 6 months of MTI, the intervention group improved in physical performance compared with the control group via Short Physical Performance Battery (SPPB) score (mean diff = 0.6, 95 % CI: 0.1, 1.0) and 8-foot up-and-go test (mean diff = −1.0 s, 95 % CI: -1.5, -0.6), and in endurance performance via 6-minute walking test (6 MW) (mean diff = 44.2 meters, 95 % CI: 17.1, 71.2). In strength performance via knee extension the intervention group improved while control group declined (mean diff = 55.0 Newton, 95 % CI: 28.4, 81.7), and also in PA (mean diff = 125.9 cpm, 95 % CI: 96.0, 155.8). Long-term effects of MTI on the particpants was assesed by estimating the mean difference in the variables measured between time-point 1 and 4: SPPB (1.1 points, 95 % CI: 0.8, 1.4); 8-foot up-and-go (−0.9 s, 95 % CI: -1.2, -0.6); 6 MW (18.7 m, 95 % CI: 6.5, 31.0); knee extension (4.2 Newton, 95 % CI: -10.0, 18.3); hand grip (6.7 Newton, 95 % CI: -4.4, 17.8); PA (−4.0 cpm, 95 % CI: -33.9, 26.0); BMI (−0.6 kg/m^2^, 95 % CI: -0.9, -0.3) and Icelandic quality of life (0.3 points, 95 % CI: -0.7, 1.4).

**Conclusions:**

Our results suggest that regular MTI can improve and prevent decline in functional fitness in older individuals, influence their lifestyle and positively affect their ability to stay independent, thus reducing the need for institutional care.

**Trial registration:**

This study was approved by the National Bioethics Committee in Iceland, VSNb20080300114/03-1

## Background

Older adults have the highest rates of disability, functional dependence and use of healthcare resources, so effective interventions for older individuals are of special interest [1,2]. Regular physical activity (PA) has many health benefits for older people, contributing to a healthy and independent lifestyle and improvements in functional capacity, quality of life, and body composition [3-5]. Regular multimodal training, based on combined endurance and strength exercise, can also minimize the physiological effects of an otherwise sedentary lifestyle by reducing the development and progression of chronic disease and disabling conditions [6,7].

Several recent multimodal training studies have focused on the detraining effect where the outcome is a loss in performance with onset as soon as six weeks after training [8-10]. Others have reported follow-up results based on multimodal training intervention and how changes in PA behavior can influence older people’s lifestyles [11-13]. However, the current literature on multimodal training studies is conflicting in regards to individual responsibility, practical knowledge, and skills. Moreover, few have investigated how multimodal training programs can influence older people’s long-term lifestyle [11-13].

To the best of our knowledge, few trials of randomized-controlled cross-over design using international recommendations with an emphasis on daily endurance and twice-a-week strength training [14], individual responsibility, practical knowledge, and skills have focused on 70–90 years old people for a 6-month extended follow-up. Therefore the main purpose of this training study was firstly to evaluate the long-term effects, 6 and 12 months following the completion of a 6-month multimodal training intervention (MTI) on functional performance, endurance performance via 6 minute walk test, strength, PA, BMI and Icelandic quality of life (IQL) in older persons, and secondly to analyze the short-term effects on outcomes after the completion of the intervention.

## Methods

### Study design

**Figure 1:**
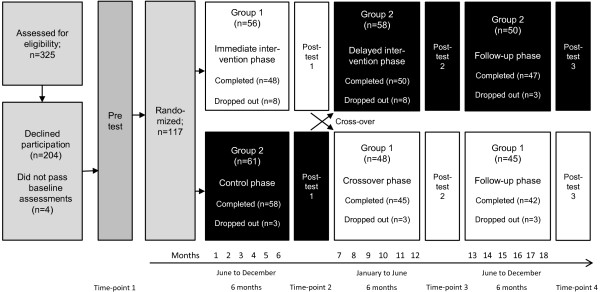
Flow of subjects through the trial.

This study was a randomized, controlled, cross-over design, performed in Reykjavik, the capital area of Iceland. The trial was conducted in four phases (time-points): 1) Enrolment and the baseline assessment, where the participants were randomized into an immediate training intervention group (Group 1) and a delayed intervention group (Group 2), 2) the immediate intervention phase, where Group 1 underwent training for 6 months and Group 2 served as a control group, 3) the crossover- and delayed intervention phase in which participants in Group 2 received the same training intervention for 6 months as Group 1 received, which from that time-point did not receive any further intervention, and 4) additional 6-month follow-up without intervention (Figure 1). Outcome assessments occurred at the end of the immediate intervention and control phase, at the end of the crossover and delayed intervention phase, and after an additional 6 months follow-up phase.

### Study participants

The participants were older individuals selected from the population-based Age, Gene/Environment Susceptibility – AGES Reykjavik Study [15] among individuals who were cognitive competent. Those who obtained a score of ≥23 points on the Mini Mental State Examination (MMSE) and ≥17 points on the Digit Symbol Substitution Test (DSST) were eligible for selection. Ninety-two of the 325 older individuals (>70), along with 25 spouses, accepted the invitation. Each participant in the trial had to fill out a questionnaire about his or her general health, and the information was reviewed by the study physician with regard to the safety of prescribed exercise. The Short Physical Performance Battery test (SPPB) [16] was also performed at screening, and a score of at least 7 points out of 12 on the test was required to be eligible for the study. The study was powered to detect a medium effect size (0.25 SD units) of any outcome measure with 80% probability, using ANCOVA to compare the post intervention measurements adjusting for baseline, not taking the clustering within groups into account. The sample size estimated was 100 at the end of the study period. We assumed a participation rate of 75% and due to the length of the study we assumed an attrition rate of about 20%.

### Physical exercise intervention

The intervention consisted of a 6-month multimodal training, with an emphasis on daily endurance training and twice-a-week strength training. This was supported by seven lectures, three on nutrition and four on healthy aging, endurance, strength and how to train.

The endurance training consisted of daily walking over the intervention phase. The duration of the training session increased progressively through the 6-month training period. During the first week, the participants trained for 20 minutes at each session, and then the duration was increased systematically over the training period with two recovery weeks. The average duration per day was estimated at 34 minutes. In the first and last eight weeks, a health instructor was on site twice a week, but in weeks 9–18, only once a week. The training took place outdoors on a 400-meter running track, except for four weeks during the winter period when the training was indoors. Other endurance training sessions were self-administered with participants following the training intervention plan from the program. A health instructor was on site once to twice-a-week, but other endurance training sessions were self-administered with participants following a training plan, using the Karvonen formula to maintain and gradually increase the intensity [17]. During the first eight weeks, the intensity level was 50% of heart rate reserve (HRR), for the next 10 weeks it was increased to 60%, and during the last eight weeks it was approximately 70% of HRR. Every participant wore a Polar heart-rate monitor to maintain his or her individual target heart rate during the training.

Resistance training took place twice-a-week, on Tuesdays and Fridays, in a fitness centre, using the circuit series strength equipment from Life Fitness (Circuit Series Strength, Brunswick Corporation, USA), always under the guidance of health instructors. The strength training consisted of 12 exercises for all major muscle groups and was individually-based following a systematic training plan. The focus was on strength-endurance training for the first 3 months but for the latter 3 months it was on strength-power. The exercises for the lower body included leg press, leg extensions and calf raises. Exercises for the upper body included bench press, chest cross, shoulder press, pull downs, biceps curls, triceps extensions, and exercises for abdominal muscles and the back. For the first 2 weeks of strength-endurance training, the training program consisted of two sets of 12 repetitions (2x12) at 50% of one repetition maximum (1RM). Every two weeks, the working load was increased by two repetitions. The strength-endurance training was done in form of circuit training program, one set at each time. In the 13^th^ week the repetitions were 18 in two sets. Recovery in form of light stretching between circuits was 3–4 minutes. In the second period, weeks 14 to 26, there program was changes from strength-endurance program to strength-power training program. The intensity went systematically from 10RM repetitions in the 14^th^ week down to 6RM in the 24^th^ week. The power training program consisted from the same 12 exercises as described before. The participants finished their exercise, two sets, with 1.5 minute rest between each set. The 9^th^ and 18^th^ week was organized as recovery weeks, with no strength training but 20 minutes endurance training every day.

### Measurements

Baseline measurements were performed over a two week period before randomization. Outcome data for Group 1 was collected at the end of the immediate intervention phase, after the completion of the 6-month crossover phase, and after a 6-month follow-up phase. Outcome data for Group 2 were collected after the control phase, after the delayed intervention phase and after the completion of a 6-month follow-up phase. Demographic and clinical data was collected by trained research staff.

The SPPB [16] was used to measure physical performance but mobility and balance were measured by the 8-foot up-and-go test [18]. Maximal isometric muscle strength of the thigh and hand on the dominant side was measured with the participant in a sitting position in an adjustable dynamometer chair (Good Strength, Metitur, Palokka, Finland) [19]. Knee extension was measured with the knee angle at 60°, the ankle fastened by a belt to a strain-gauge system and with the participant’s hands gripping the edge of the seat. Handgrip strength was measured with a dynamometer fixed to the arm of the same chair with the elbow flexed at 90°, using the same instructions and methods as for the lower limbs. Endurance performance was measured using the 6-minute walk test (6 MW) according to a standardized protocol [20]. The heart rate of participants was measured before and directly after completing the walk, and once more one minute later.

Total PA was assessed with Actigraph accelerometers (AG; Model 7164, version 2.2; ActiGraph Health Services, Fort Walton Beach, Florida, USA), which were programmed to record PA over one-minute intervals (60s epoch) [21]. The accelerometers were worn on the hip for six consecutive days, four week days and two weekend days, from the time the participant woke up until he or she went to sleep. Only data from monitors worn a minimum of eight hours per day, for at least two weekdays and one weekend day were included in the analysis. Average counts per minute (cpm) for these days measured by the accelerometer were calculated for each participant and were used to estimate PA level. A questionnaire was also used to estimate PA behavior in a typical week at each measurement time-point. During the training period, each participant had a 6-month intervention diary in which he or she had notes about suggested training regimens, but also confirmed their daily PA behavior as time spent on walking and strength training. The questionnaire and participant’s diary were based on a Global Physical Activity Questionnaire [22].

Standing height was measured to the nearest 0.1 cm with a portable stadiometer (Seca 206, Seca Ltd, Birmingham, UK). Body weight was determined to the nearest 0.1 kg using a calibrated scale (Seca HV120, Seca Ltd, Birmingham, UK) with the participant in light clothing. Body mass index (BMI) was calculated as body mass (kg) divided by height squared (m^2^).

The health-related quality of life (HRQL) was measured with a validated generic Icelandic instrument, Icelandic Quality of Life questionnaire (IQL). The IQL-test has norms for males and females in different age groups in order to evaluate individual deviation in HRQL. Five factors explain two thirds of the variance: general health (23.4%), mental well-being (20.5%), satisfaction (9.0%), sleep (6.9%), and finance (6.3%) [23].

### Statistical analysis

Difference in each outcome at baseline and progression over time was analyzed using a repeated measures model with a first-order autoregressive covariance structure. A parameter was included in the model to represent each time-point for the immediate group (I), Group 1, and the delayed intervention group (D), Group 2: μ_I1_, μ_I2_, μ_I3_, μ_I4_, μ_D1_, μ_D2_, μ_D3_, μ_D4_. An adjustment was made for age and sex. The mixed models method allows for missing values in the response. All participants had at least a baseline measure and a measure after the intervention. Participants with missing values at other time–points were included in the analysis. Contrasts between time-points were estimated from linear combinations of the model parameters. For example: The difference between groups at baseline was estimated as μ_I1_ – μ_D1_; the immediate intervention effect was estimated as μ_I2_ – μ_I1_; the change between the repeat baseline and the baseline for the delayed intervention group was estimated as μ_D2_ – μ_D1_; the delayed intervention effect as μ_D3_ – μ_D2_; the overall intervention effect as (μ_I2_ – μ_I1_ + μ_D3_ – μ_D2_)/2; and the overall improvement completed follow-up phase by both groups (the difference between time-point 4 and time-point 1) as (μ_I4_ – μ_I1_ + μ_D4_ – μ_D1_)/2. The results were generated using the SAS MIXED model procedure in SAS/STAT software, version 9.2.

## Results

### Baseline characteristics and dropout

A diagram of subjects’ flow through this randomized cross-over trial detailing the measurement phases is illustrated in Figure 1. Out of the 325 who were potentially eligible, 121 (37%) accepted the participation. The major reason for refusing participation in the study was too long and binding periods, not interested or because of spouse illness. Four participants out of the 121 did not pass the baseline assessments of the study. Thus, 117 subjects were randomized to the immediate intervention group (Group 1; n = 56) and delayed intervention group (Group 2; n = 61). A total of 48 subjects, 85.7% of those randomized for Group 1, completed the immediate intervention phase, and a total of 50 subjects, 82% of those randomized for Group 2, completed the delayed intervention. Overall, 98 subjects out of the 117 who were randomized received the entire 6-month training intervention. Reasons for attrition included spouse’s illness or lack of time due to commitment to family. Significant differences at baseline characteristics were seen in age between the 98 subjects who completed the 6-month MTI (78.9 ± 4.5) and the 19 subjects who were randomized but did not complete the intervention (82.6 ± 3.5), in 8-foot up-and-go test (6.3 ± 1.2 seconds by MTI vs 7.4 ± 1.3 seconds; *p* < .01), and in strength, knee-extension (340.6 ± 94.3 Newton by MTI vs 273.6 ± 70.9 Newton; *p* < .05).

The baseline data for the characteristics of the study subjects randomized to Group 1 and Group 2 are summarized in Table 1. The mean age was approximately 80 and the range 71–90. The only significant baseline difference between the two groups was in age, 80.8 ± 4.7 in Group 1 vs 78.3 ± 4.1 in Group 2.

**Table 1:** Baseline characteristics of subjects randomized to immediate intervention (Group 1) and delayed intervention (Group 2)

	**Immediate intervention**	**Delayed intervention (Control)**	**Difference between**
	**Group 1**	**Group 2**	**groups at baseline**
**Characteristic**	**(*n*) Mean±SD (Range)**	**(*n*) Mean±SD (Range)**	***p*-value**
Age	(56) 80.8±4.7 (73–90)	(61) 78.3±4.1 (71–88)	.003
Male (age)	(25) 81.9±4.8 (75–90)	(29) 79.0±4.3 (71–88)	.024
Female (age)	(31) 79.9±4.6 (73–89)	(32) 77.8±3.8 (72–85)	.045
Physical performance			
SPPB (points)	(56) 10.1±1.5 (7–12)	(61) 10.0±1.3 (7–12)	.168
Balance (points)	(56) 3.3±0.8 (2–4)	(61) 3.2±0.9 (1–4)	.167
Walk (seconds)	(56) 3.7±0.9 (2.3–8.4)	(61) 3.6±0.5 (2.7–4.9)	.549
Chair (seconds)	(56) 12.8±2.5 (7.7–18.0)	(61) 13.2±2.6 (8.4–20.0)	.177
8 foot up-and-go (seconds)	(56) 6.4±1.4 (4.4–13.2)	(61) 6.5±1.1 (4.4–9.7)	.217
Strength performance			
Knee extension (Newton)	(56) 328.4±96.3 (127.1–547.5)	(61) 330.9±92.5 (150.0–585.9)	.340
Hand grip (Newton)	(56) 311.0±96.8 (168.4–567.0)	(61) 341.9±108.4 (132.9–619.4)	.193
Endurance performance			
Six min walking (meter)	(56) 450.0±84.0 (255.0–656.0)	(61) 459.8±64.8 (300.0–612.0)	.592
Physical activity (cpm)	(39) 258.8±122.9 (100.0–589.0)	(44) 253.9±101.8 (106.0–537.0)	.275
BMI (kg/m^2^)	(56) 27.6±5.3 (20.6–45.9)	(61) 27.4±3.4 (20.1–36.3)	.406
Icelandic Quality of Life (points)	(54) 55.7±5.5 (40–64)	(57) 55.9±5.1 (38–63)	.574

Values are shown as numbers in groups (*n*), means with standard deviation (SD) and range.

SD = Standard deviation.

SPPB = Short physical performance battery.

cpm = Average counts per minute.

BMI = Body mass index.

### The immediate intervention phase and the control phase

The results of the immediate intervention phase of the trial are presented in Table 2. There was a significant difference between the intervention and control groups in the changes for physical performance including better overall scores for the SPPB (mean diff = 0.6, *p* < .05) and chair rises (mean diff = −1.8 s, *p* < .001), in mobility and balance by the 8-foot up-and-go test (mean diff = −1.0 s, *p* < .001), in knee extension strength (mean diff = 55.0 Newton, p < .001) and in endurance by the 6-minute walking test (mean diff = 44.2 m, *p* < .001). There were also significant increases in daily PA (mean diff = 125.9 cpm, *p* < .001) between the groups where the immediate intervention group increased their PA around 13% while at the same time the delayed intervention group showed a 14% decrease. Significant changes between baseline and MTI within the immediate intervention group was seen on all measurements apart from balance part in the SPPB and IQL.

**Table 2:** Outcomes for subjects who completed the immediate intervention phase and the control phase, and between-group differences

**Group 1 (Immediate intervention phase) (*n*=48)**				**Group 2 (Control phase) (*n*=58)**			**Between-group**
**Outcome and Values**	**Baseline**	**MTI**	**Change**	**Baseline**	**Rep. Base**	**Change**	**difference**
	**Mean (SE)**	**Mean (SE)**	**Diff in means (95% CI)**	**Means (SE)**	**Means (SE)**	**Diff in means (95% CI)**	**Diff in means (95% CI)**
Physical performance							
SPPB (points)	10.1 (0.2)	10.7 (0.2)	0.6 (0.2 to 0.9)***	9.8 (0.2)	10.1 (0.2)	0.3 (0.0 to 0.6)	0.6 (0.1 to 1.0)*
Balance (points)	3.4 (0.1)	3.4 (0.1)	0.1 (-0.2 to 0.3)	3.2 (0.1)	3.2 (0.1)	0.1 (-0.2 to 0.3)	0.2 (-0.1 to 0.5)
Walk (seconds)	3.7 (0.1)	3.3 (0.1)	-0.3 (-0.5 to -0.2)***	3.6 (0.1)	3.4 (0.1)	-0.2 (-0.3 to -0.1)**	-0.1 (-0.2 to 0.1)
Chair (seconds)	12.7 (0.3)	11.0 (0.3)	-1.7 (-2.2 to -1.2)***	13.3 (0.3)	12.8 (0.3)	-0.5 (-1.0 to -0.1)*	-1.8 (-2.7 to -0.8)***
8 foot up-and-go (seconds)	6.3 (0.2)	5.7 (0.2)	-0.6 (-0.9 to -0.3)***	6.6 (0.2)	6.7 (0.2)	0.1 (-0.2 to 0.4)	-1.0 (-1.5 to -0.6)***
Strength performance							
Knee extension (Newton)	338.8 (9.3)	367.3 (9.7)	28.5 (15.3 to 41.7)***	326.2 (9.1)	312.3 (9.2)	-13.9 (-26.0 to -1.9)*	55.0 (28.4 to 81.7)***
Hand grip (Newton)	323.1 (8.6)	334.4 (8.8)	11.3 (1.4 to 21.2)*	339.0 (8.4)	343.1 (8.5)	4.1 (-4.8 to 13.1)	-8.8 (-33.1 to 15.6)
Endurance performance							
Six min walking (meter)	457.0 (9.6)	491.1 (9.8)	34.2 (23.3 to 45.0)***	449.7 (9.3)	447.0 (9.4)	-2.7 (-12.5 to 7.1)	44.2 (17.1 to 71.2)**
Physical activity (cpm)	272.9 (16.9)	307.1 (15.7)	34.2 (0.8 to 67.6)*	247.0 (16.1)	211.7 (15.0)	-35.2 (-66.2 to -4.3)*	125.9 (96.0 to M155.8)***
BMI (kg/m^2^)	27.7 (0.6)	27.3 (0.6)	-0.5 (-0.7 to -0.3)***	27.0 (0.6)	26.9 (0.6)	-0.2 (-0.4 to 0.0)	0.4 (-1.2 to 2.0)
Icelandic quality of life (points)	55.8 (0.7)	56.6 (0.7)	0.7 (-0.2 to 1.7)	55.3 (0.7)	54.6 (0.7)	-0.6 (-1.5 to 0.3)	1.9 (-0.1 to 3.9)

Values are shown as means with standard error (SE) at following time points: baseline and MTI by Group 1 and baseline and repeated baseline by Group 2, 95% confidence interval in means (95% CI) comparing changes between groups, and significant differences; * *p* < .05, ** *p* < .01, *** *p* < .001.

MTI = Multimodal Training Intervention.

Rep. Base = Repeat Baseline.

SE = Standard error.

SPPB = Short physical performance battery.

cpm = Average counts per minute.

BMI = Body mass index.

### The crossover and delayed intervention phase

The results of the crossover phase of the trial are presented in Table 3. Group 2 had improvements in their delayed intervention (Table 3, column 6) comparable to the immediate intervention of Group 1 (Table 2, column 3). In addition, all gains seen in the immediate intervention by Group 1 were maintained over the following 6-month period where there was no formal training on behalf of the health educators (Table 3, column 3).

**Table 3:** Outcomes for subjects who completed the crossover and delayed intervention phase, and between-group differences

**Group 1 (No Intervention) (*n*=45)**				**Group 2 (Delayed Intervention) (*n*=50)**			**Between-group**
**Outcome and Values**	**MTI**	**Crossover**	**Change**	**Rep. Base**	**Delayed MTI**	**Change**	**difference**
	**Mean (SE)**	**Mean (SE)**	**Diff in means (95% CI)**	**Means (SE)**	**Means (SE)**	**Diff in means (95% CI)**	**Diff in means (95% CI)**
Physical performance							
SPPB (points)	10.7 (0.2)	11.0 (0.2)	0.3 (0.0 to 0.7)	10.1 (0.2)	10.7 (0.2)	0.6 (0.2 to 0.9)***	−0.3 (−0.8 to 0.2)
Balance (points)	3.4 (0.1)	3.5 (0.1)	0.0 (−0.2 to 0.3)	3.2 (0.1)	3.4 (0.1)	0.1 (−0.1 to 0.4)	−0.1 (−0.4 to 0.2)
Walk (seconds)	3.3 (0.1)	3.3 (0.1)	0.0 (−0.2 to 0.1)	3.4 (0.1)	3.3 (0.1)	−0.1 (−0.2 to 0.1)	0.0 (−0.2 to 0.3)
Chair (seconds)	11.0 (0.3)	10.4 (0.3)	−0.6 (−1.1 to −0.1)*	12.8 (0.3)	11.5 (0.3)	−1.3 (−1.8 to −0.8)***	1.0 (0.1 to 2.0)*
8 foot up-and-go (seconds)	5.7 (0.2)	5.6 (0.2)	−0.1 (−0.4 to 0.2)	6.7 (0.2)	6.0 (0.2)	−0.7 (−1.0 to −0.4)***	0.4 (−0.1 to 0.9)
Strength performance							
Knee extension (Newton)	367.3 (9.7)	355.7 (9.9)	−11.6 (−25.0 to 1.7)	312.3 (9.2)	343.6 (9.6)	31.3 (18.6 to 44.0)***	−12.1 (−39.4 to 15.2)
Hand grip (Newton)	334.4 (8.8)	335.4 (9.0)	1.1 (−9.0 to 11.1)	343.1 (8.5)	357.5 (8.7)	14.3 (4.9 to 23.8)**	22.0 (−2.9 to 46.9)
Endurance performance							
Six min walking (meter)	491.1 (9.8)	481.1 (10.0)	10.0 (−21.3 to 1.3)	447.0 (9.4)	462.8 (9.7)	15.8 (5.3 to 26.3)**	−18.3 (−46.0 to 9.5)
Physical activity (cpm)	307.1 (15.7)	277.1 (16.4)	−30.1 (−60.7 to 0.6)	211.7 (15.0)	337.6 (16.0)	125.9 (96.0 to 155.8)***	60.6 (15.1 to 106.0)**
BMI (kg/m^2^)	27.3 (0.6)	27.3 (0.6)	0.0 (−0.2 to 0.2)	26.9 (0.6)	26.4 (0.6)	−0.5 (−0.7 to −0.3)***	−0.9 (−2.5 to 0.8)
Icelandic quality of life (points)	56.6 (0.7)	56.2 (0.7)	−0.4 (−1.4 to 0.6)	54.6 (0.7)	55.5 (0.7)	0.9 (−0.1 to 1.8)	−0.6 (−2.7 to 1.4)

Values are shown as means with standard error (SE) at following time points; MTI and crossover by Group 1 and repeated baseline and delayed MTI by Group 2, 95% confidence interval in means (95% CI) comparing changes between groups, and significant differences; * *p* < .05, ** *p* < .01, *** *p* < .001.

MTI = Multimodal Training Intervention.

Rep.Base = Repeat Baseline.

SE = Standard error.

SPPB = Short physical performance battery.

cpm = Average counts per minute.

BMI = Body mass index.

### Multimodal training intervention phase by both groups together

The effects of MTI in all 98 subjects in both Group 1 and Group 2 who completed the intervention are pooled together and summarized in Table 4. All changes in all measurements were statistically significant except for the balance test in SPPB. This may have represented a ceiling effect because approximately 58% of the subjects obtained 4 points or the maximum results from this test.

**Table 4:** Outcomes for all subjects who completed MTI in both groups

		**Completed MTI (*n*=98)**	
**Outcome and Values**	**Baseline Repeated baseline**	**Immediate MTI Delayed MTI**	**Change**
	**Means (SE)**	**Means (SE)**	**Diff in means (95% CI)**
Physical performance			
SPPB (points)	10.2 (0.1)	10.7 (0.1)	0.6 (0.3 to 0.8)***
Balance (points)	3.3 (0.1)	3.4 (0.1)	0.1 (−0.1 to 0.3)
Walk (seconds)	3.5 (0.1)	3.3 (0.1)	−0.2 (−0.3 to −0.1)***
Chair (seconds)	12.6 (0.2)	11.1 (0.2)	−1.5 (−1.8 to −1.1)***
8 foot up-and-go (seconds)	6.4 (0.1)	5.7 (0.1)	−0.6 (−0.8 to −0.4)***
Strength performance			
Knee extension (Newton)	332.2 (7.2)	360.8 (7.2)	28.6 (18.7 to 38.5)***
Hand grip (Newton)	336.0 (6.4)	349.0 (6.4)	13.0 (5.8 to 20.2)***
Endurance performance			
Six min walking (meter)	457.5 (7.5)	482.4 (7.5)	24.9 (17.2 to 32.6)***
Physical activity (cpm)	240.3 (12.2)	326.0 (11.6)	85.6 (62.2 to 109.1)***
BMI (kg/m^2^)	27.3 (0.5)	26.8 (0.5)	−0.46 (−0.6 to −0.3)***
Icelandic quality of life (points)	55.6 (0.5)	56.4 (0.5)	0.8 (0.1 to 1.5)*

Values are shown as means with standard error (SE) at following time points pulled together: baseline by Group 1 and repeated baseline by Group 2, and immediate MTI by Group 1 and delayed MTI by Group 2, and as 95% confidence interval in means (95% CI) comparing changes for all subjects who completed MTI, and significant differences; * *p* < .05, ** *p* < .01, *** *p* < .001.

MTI = Multimodal training intervention.

SE = Standard error.

SPPB = Short physical performance battery.

cpm = Average counts per minute.

BMI = Body mass index.

**Figure 2:**
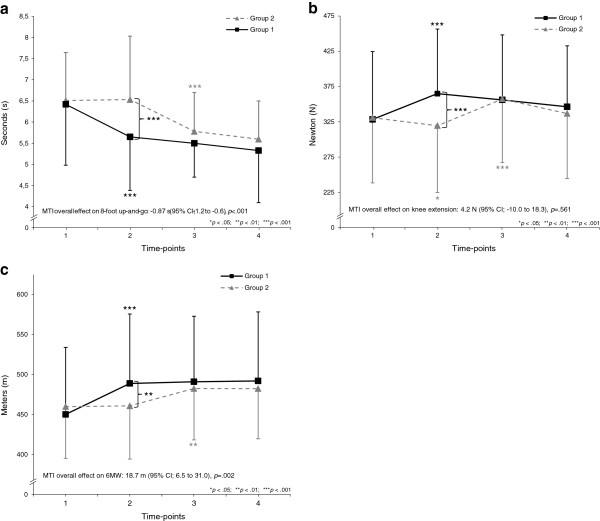
**a Outcome measures from Group 1 and Group 2 in the 8-foot up-and-go test at four time-points: At baseline (1); after 6-month immediate intervention and control phase (2); after completed crossover phase and delayed intervention phase (3); after completed follow-up 2 phase by Group 1 and follow-up 1 phase by Group 2 (4).2b** Outcome measures from Group 1 and Group 2 in knee-extension strength performance test at four time-points: At baseline (1); after 6-month immediate intervention and control phase (2); after completed crossover phase and delayed intervention phase (3); after completed follow-up 2 phase by Group 1 and follow-up 1 phase by Group 2 (4). **2c** Outcome measures from Group 1 and Group 2 in 6 MW endurance performance test at four time-points: At baseline (1); after 6-month immediate intervention and control phase (2); after completed crossover phase and delayed intervention phase (3); after completed follow-up 2 phase by Group 1 and follow-up 1 phase by Group 2 (4).

Figure 2a–c demonstrates outcome measures at four time-points and MTI overall effect from both groups in long-term improvements in 8-foot up-and-go (mean diff = −0.9, *p* < .001) and 6 MW (mean diff = 18.7 m, *p* < .01), but strength performance measured as knee extension maintained (mean diff = 4.2 Newton, *p* > .05) compared to baseline. MTI overall effects in other measurements was following: Balance within SPPB (mean diff = 0.2 s, *p* < .05), 4 m walking within SPPB (mean diff = −0.7 s, *p* < .001), chair rises within SPPB (mean diff = −2.7 s, *p* < .001), hand grip (mean diff = 6.7 Newton, *p* > .05), IQL (mean diff = 0.3, *p* > .05), daily PA (mean diff = −4.0 cpm, *p* > .05) and BMI (mean diff = −0.6, *p* < .001).

### Physical activity behavior

**Figure 3:**
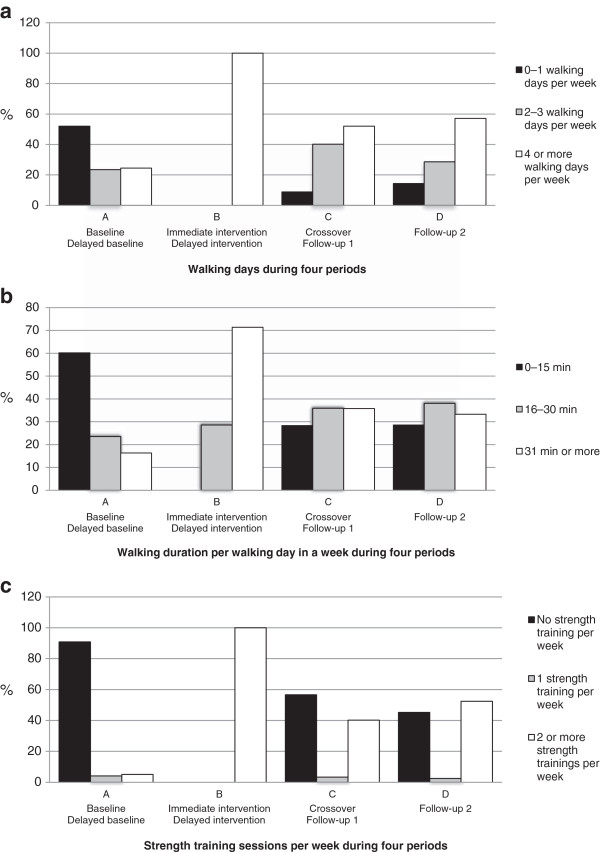
**a Distribution of participation spent in physical activity, walking days in a week (%), during following four periods for both groups: During the period before baseline and delayed baseline (A), during immediate intervention and delayed intervention (B), during crossover and follow-up 1 (C) and during follow-up 2 by Group 1 (D).3b** Distribution of participation spent in physical activity, walking duration per walking day in a week (%), during following four periods for both groups: During the period before baseline and delayed baseline (A), during immediate intervention and delayed intervention (B), during crossover and follow-up 1 (C) and during follow-up 2 by Group 1 (D). **3c** Distribution of participation spent in physical activity, strength training sessions per week (%), during following four periods for both groups: During the period before baseline and delayed baseline (A), during immediate intervention and delayed intervention (B), during crossover and follow-up 1 (C) and during follow-up 2 by Group 1 (D).

Figure 3a–c illustrates distribution of time spent in daily PA behavior (%) in terms of walking-days, walking-duration and strength training sessions per week by both groups together during four periods; the period before baseline by Group 1 and delayed baseline by Group 2 (period A); during the immediate intervention by Group 1 and delayed intervention by Group 2 (period B); during the crossover by Group 1 and follow-up 1 by Group 2 (period C); and during follow-up 2 by Group 1 (period D). During period A, approximately half of the participants had none or just one walking day per week and about 60% estimated that their walking duration per session was less than 15 minutes. The participation in strength training during this period was about 10%. Period B shows the PA behavior over the 6-month immediate and delayed intervention. During period C, 90% of the participants reported that they had two or more walking days per week, 72% said that they spent from 16 and up to 75 minutes in every walking session, and 43% said they performed strength training on a regular basis, generally twice a week. Comparable outcomes during period D in walking days and minutes in walking sessions were measured by Group 1, but 55% of the group informed that they participated in strength training on a regular basis.

## Discussion

This study resulted in notable significant improvements in functional performance, strength, endurance via 6 MW, PA, BMI and IQL among older individuals. At the crossover phase and follow-ups they retained their improvements above baseline status despite some attenuation in strength, IQL and PA. Furthermore, substantially positive changes were seen in participant’s lifestyle changes in daily PA behavior, both in endurance and strength training at crossover and follow-up phases. Results from this study clearly demonstrate that well organized longitudinal multimodal training intervention can improve several physiological as well as psychological factors for relatively long periods among old people.

Age-related trends within studies were remarkably similar and even though the sexes differ in levels of physical fitness, the observed age differences was similar within a given population [24]. The effects from the training intervention in our study showed statistically improvements in functional performance, endurance via 6 MW and strength, PA, BMI and IQL. Similar positive health-related changes have been shown in several other studies [3,4,10-13].

In this study the participants were older and the training intervention and follow-up periods was longer than in most existing multimodal studies of PA that we compare to [4,8,9]. Our training design very clearly met the minimum standards recommended in guidelines for older individuals, both in the endurance and strength parts of the study [6,25]. Generally, other multimodal training studies satisfied the strength part of recommendations, but not the endurance part, which results in smaller comprehensive improvements [10,11,13,26]. For example, the results from body composition in our study showed that older subjects were able to achieve a decrease in BMI after six months of training, but at the same time enhance their strength. Similar results between this study and the findings of others were seen for gait speed, where functional decline was observed one month after the cessation of training [11].

The frequency, duration, and intensity employed in this study may have contributed to the improvement in 6 MW after the MTI. But the reason for the maintenance at crossover and follow-ups measurements lies arguably in lifestyle changes and self-organized training by the participants after the intervention period. A study [10] with training sessions twice per week showed clearly that this is not enough stimulus for measurable improvements as was evident in our study. Our intervention methods, with about 240 minutes per week of moderate-intensity exercise for six months, met the recommended 150 minutes per week of moderate-intensity aerobic activity, for the elderly [6].

These results also underline the importance for older people to participate in regular training for their quality of life [12]. To the knowledge of the authors of the current study, few studies were available where 6-month multimodal training with 6- and 12-month follow-ups has been performed for this age group. In most prior studies, the participants were younger, had significantly worsened at the follow-up measurements, and were even worse after one year of follow-up compared to their baselines [9,10]. Overall MTI outcomes in our study generally remained statistically better compared to baseline, and none were statistically lower. These long-term positive results are likely to have three main reasons. First, the use of reasonable and progressive training protocol with a desirable balance between the appropriate volume and intensity of the training sessions throughout the whole intervention period. Secondly, the ability of the participants to follow the main goals of this study: to stay independent and carry on with the PA after the MTI, and finally, the guidance part by health instructors, both in educating and encouraging the participants in their work. The validity of the last point needs further examination. In addition, the exercise program after MTI could be continued by the participants with less support from a health instructor. Instead of twelve to sixteen exercise sessions with a health instructor per month, we would recommend, based on our findings, two to four sessions with a health instructor per month, in addition, independent PA, to maintain endurance and strength.

This multimodal training intervention study had 6 and 12 months follow-up time-points. The results clearly demonstrated that this multimodal training program improved endurance as well as strength performance, decreased BMI and increased and maintained IQL in older individuals for a relatively long period of time. Hence, this type of training could have a clinically relevant impact on older individuals in the general population if applied to a large number of individuals. The use of educated health instructors during the training intervention and working closely with the people might help to maintain their performance after the formal training period. Such implementation seems to motivate and support older individuals who seek to maintain their physical health and IQL on their own over a long period of time. This was strongly supported by the observation that about 60% of the participants estimated that their walking duration per session was less than 15 minutes before they entered the study. On the other hand, about 90% had two to seven walking days per week, whereof over 70% said that they spent from 16 and up to 75 minutes in every walking session for up to a year after the training intervention.

This study had several strengths that address some of the limitations of previous multimodal training studies. Our objective was to influence participants’ lifestyles and everyday activities during the MTI, with a focus on individual responsibility and to prepare the participants to train independently after completing the immediate or the delayed intervention phase. Our training was based on international recommendations [27] and the methods and philosophy were similar to those that would be used in a sedentary population, with few allowances for age. The use of accelerometers to assess physical activity volume and intensity and the low dropout rate for this age-group can be classified as strength of this study.

## Conclusions

In conclusion, this study clearly demonstrates that multimodal training intervention based on endurance and strength exercise is feasible and beneficial in older populations, particularly among those who have not been physically active before. Our results suggest that regular MTI can affect and improve long-term retention, 12 and 18 months after the baseline measurements, of functional fitness and endurance performance measured by 6 MW and maintain strength performance and quality of life in old people. In addition, the intervention can influence the lifestyle behavior concerning strength and endurance training. Therefore, we suggest that regular MTI can prevent decline in functional fitness in old people, influence their lifestyle and positively affect their ability to stay independent; thus reducing the need for institutional care. For societies and individual health practitioners it is therefore important to encourage all older persons to increase their PA and give them opportunities to participate in a supervised multimodal training program.

## Abbreviations

PA: Physical activity; MTI: Multimodal training intervention; SPPB: Short Physical Performance Battery; IQL: Icelandic quality of life; HRQL: The health-related quality of life; BMI: Body mass index; 6 MW: 6-minute walk test.

## Competing interests

The authors declare that they have no competing interest.

## Authors’ contributions

All authors were involved in the study concept and design, and in obtaining funding. JG, EJ, and VG were involved in the acquisition of data, and JG, EJ, SAA, ASO, TH, KS, VG, TA, and PVJ were involved in analysis and interpretation of data. All authors drafted the manuscript and critically revised it for important intellectual content. All authors read and approved the final manuscript.
